# Tongue function and its influence on masticatory performance in patients treated for oral cancer: a five-year prospective study

**DOI:** 10.1007/s00520-019-04913-y

**Published:** 2019-07-04

**Authors:** Reilly J. de Groot, Matthias A.W. Merkx, Merel N.S. Hamann, Henk S. Brand, Anton F.J. de Haan, Antoine J.W.P. Rosenberg, Caroline M. Speksnijder

**Affiliations:** 1 Department of Oral and Maxillofacial Surgery and Special Dental Care, University Medical Center Utrecht, Utrecht University, 3508 GA Utrecht, The Netherlands; 2 Department of Oral and Maxillofacial Surgery, Radboud University Medical Center, Nijmegen, The Netherlands; 3 Academic Centre for Dentistry Amsterdam (ACTA), University of Amsterdam and Vrije Universiteit Amsterdam, Amsterdam, the Netherlands; 4 Department for Health Evidence, Section Biostatistics, Radboud University Medical Center, Nijmegen, The Netherlands; 5 University Medical Center Utrecht, Julius Center for Health Sciences and Primary Care, Utrecht University, Utrecht, The Netherlands; 6 University Medical Center Utrecht, Cancer Center, Department of Head and Neck Surgical Oncology, Utrecht University, Utrecht, The Netherlands

**Keywords:** Masticatory performance, Mixed model analysis, Tongue function, Prospective study, Oral cancer

## Abstract

**Purpose:**

The purpose of this study was to observe the impact of oral oncological treatment, including the recovery of several tongue functions (force, mobility, and sensory functions), and to determine the influence of these functions on masticatory performance.

**Materials and methods:**

Masticatory performance and tongue force, mobility, and sensory functions were determined in 123 patients with oral cavity cancer. The assessments were performed 4 weeks before treatment and 4 to 6 weeks, 6 months, 1 year, and 5 years after treatment. Generalized estimation equations and mixed model analyses were performed, correcting for previously identified factors in the same population.

**Results:**

A significant deterioration in tongue mobility and sensory function was observed in patients with mandible and tongue and/or floor-of-mouth tumors. Better tongue force and sensory function (thermal and tactile) positively influenced masticatory performance, and this effect was stronger where fewer occlusal units were present. The effect of both the tongue force and maximum bite force was weaker in dentate patients in comparison with patients with full dentures. A web-based application was developed to enable readers to explore our results and provide insight into the coherence between the found factors in the mixed model.

**Conclusions:**

Tongue function deteriorates after oral oncological treatment, without statistically significant recovery. Adequate bite and tongue forces are especially important for patients with a poor prosthetic state. Patients with sensory tongue function deficits especially benefit from the presence of more occluding pairs.

## Introduction

Mastication is a coordinated process, integrating central control, sensory input, and muscle function. Food is introduced to the mouth in bite-sized pieces, positioned on the occlusal surfaces by the cheek and tongue, and pulverized by chewing. Finally, it is collected in the oropharynx to form a bolus, ready for swallowing [1]. Both sensory tongue function for food bolus allocation and motor function for food bolus transportation are essential for effective masticatory performance [2]. When, for example, the laterodeviation to the left of the tongue is impeded, mastication on that side can be impaired by the hampered transportation of food to the occlusal surfaces [3].

Masticatory performance significantly decreases after oral cancer treatment, and recovery generally occurs between 1 and 5 years after treatment [4]. Factors influencing masticatory performance in patients with oral cancer are their maximum bite force (MBF), maximum mouth opening (MMO), tumor location, dental state, number of occluding pairs, and the time since treatment [4]. Scarring or radiation fibrosis can reduce or even inhibit tongue mobility (TM) [5, 6], while lingual and/or hypoglossal nerve resection can lead to the loss of sensory and motor functions in the tongue [7]. This deterioration in tongue function can be limited by primary surgical reconstruction after a glossectomy, including the primary closure, split skin grafting, the use of local flaps, or a microvascular-free flap, which is functionally favorable over any secondary procedure [8, 9]. It is possible that the importance of several tongue functions related to masticatory performance might increase as the dental state declines. However, as edentulous patients tend to break down soft food particles between the tongue and palate, these patients also rely on adequate functioning of the tongue [6].

Before we can understand the importance of tongue function for the masticatory performance of patients confronted with oral cancer, tongue function must be quantitatively evaluated over time. The purpose of this prospective cohort study was therefore to observe the longitudinal results of TM, sensory function, and tongue force in patients treated for oral cancer. Secondly, the impacts of tongue functions on masticatory performance were studied, together with the influence of dental state on this effect.

## Materials and methods

### Patients

Patients with oral cancer were recruited between January 2007 and August 2009 at the University Medical Center Utrecht (UMC Utrecht) and Radboud University Medical Center (Radboudumc) in Nijmegen. These patients had three groups of tumor locations: (1) maxilla, (2) mandible, and (3) tongue and/or floor of the mouth (TFM). The patients were treated at least surgically, with a curative intent [4, 10]. Adjuvant radiotherapy was administered according to the Dutch Head and Neck Society Guidelines. Patients were excluded from the study if they underwent radiotherapy as a primary treatment or if they had had another previous and/or concurrent malignancy, had a cognitive impairment, or were unable to understand Dutch. The experimental protocol used was in accordance with the Declaration of Helsinki and was approved by the Ethics Committees of the UMC Utrecht and Radboudumc. All patients signed informed consent.

### Assessments

The patients were assessed at a maximum of 4 weeks prior to their primary treatment (t_0_); 4 to 6 weeks after their surgery and/or radiotherapy (respectively, t_1a_ and t_1b_); and 6 months (t_2_); 1 year (t_3_); and 5 years (t_4_) after their primary treatment. Their baseline demographics, as well as details on their disease, treatment, and reconstruction, were extracted from their medical records. At each assessment stage, the presence and type of prostheses in either jaw (edentulous, full prosthesis, implant-supported prosthesis, dentate), number of occluding pairs, masticatory performance, MBF, tongue force, TM, and sensory functions of the tongue (thermal and tactile) were assessed.

### Masticatory performance

Masticatory performance was assessed using the mixing ability test [11, 12]. The subjects performed 20 chewing strokes on a two-colored (red and blue) room-temperature wax tablet, after which the tablet was flattened and the degree of color-mixing was digitally assessed using specialized software. The Mixing Ability Index (MAI) ranged from 0 to 30 (30 being the worst score possible), based on the spread of the color intensities in the combined image of both sides of the tablet. For patients with oral cancer, a MAI below 20 likely indicates a subject able to chew all food types, subjects scoring between 20 and 24 should be able to chew soft food types, and those scoring over 25 would be expected to have difficulty with both solid and soft food types [5, 12–14].

### Maximum tongue force

Maximum tongue force (MTF) was measured twice in a vertical plane using a device consisting of a unilateral strain gauge mounted on a mouthpiece. To measure tongue force, the strain gauge element (surface 100 mm^2^, height 4.5 mm) was positioned between the tongue and the palate at the midline of the tongue, 5 mm from the tip. The highest tongue force of both efforts was used as the MTF [6].

### Tongue mobility

To measure TM, the subjects were asked to protrude and laterodeviate (left and right) their tongue as far as possible. Their TM was rated on a three-point scale, with the following possible scores for protrusion/laterodeviation: 2, cannot reach the lower lip/mouth corner; 1, reaches the lower lip/mouth corner; and 0, reaches beyond the lower lip/mouth corner [6]. The three variables (left and right laterodeviation and protrusion) were recoded into a single variable, selecting the single highest (thus worst) score of the three as the outcome for general tongue function to maintain the clinical applicability of the results. The results were thus categorized into the following three-point scale: 0, normal mobility; 1, impaired mobility; and 2, no mobility [6].

### Sensory function

Tongue thermal and tactile sensory functions (SF_thermal_ and SF_tactile_, respectively) were assessed by presenting the subjects with three pairs of real and fake stimuli in a random order [15, 16]. The magnitude of the test stimulus was chosen as the value at which healthy subjects could just detect this stimulus with almost no errors, enabling the patients to consistently make the correct choice at uninjured sites. This was performed on the left and right side of the tongue, 10 mm from both the tip and the lateral border of the tongue. The patients’ eyes remained shut and they reported which time the real stimulus was applied [6]. SF_thermal_ was tested using a heat-conducting aluminum bar (2.0-mm diameter) and a non-heat–conducting acrylic bar, both at 22 °C, as real and fake stimuli, respectively. SF_tactile_ was tested using a Semmes-Weinstein monofilament (Semmes–Weinstein Aesthesiometer, Stoelting Co., Wood Dale, IL, USA) with an index number of 3.22. In this case, the real stimulus was a touch with the filament, and for the fake stimulus, the filament was turned away [6]. For both functions, the sum of the outcomes on the left and right side was interpreted as 0, unimpaired; 1, unilateral impairment; and 2, bilateral impairment.

### Maximum bite force

The MBF was assessed using a strain gauge (surface 100 mm^2^, height 2.8 mm) between the first molars (or molar region). The subject was asked to bite two times, as hard as possible, on both the left and right sides. The mean of the highest left and right bite forces was taken as the MBF [4, 10].

### Maximum mouth opening

MMO was assessed using an extra-oral protocol in which the distance between two stickers on the nose and chin was measured with a digital caliper. The distance was measured twice with the mouth closed, twice with the lips closed but with no molar contact, and twice with the mouth fully open. The mean of the two closed assessments was subtracted from the highest “open” position and presented as the MMO [4, 10].

#### Statistical analysis

The MTF at assessment moments t_1a_ and t_1b_ (representing patients who underwent only surgery and those who also underwent radiotherapy, respectively, 4 to 6 weeks after their surgery) were compared using a paired *t* test, while a Wilcoxon signed rank test was used to assess the TM and SF at these assessment moments. No significant differences were found for any of these variables, so t_1a_ and t_1b_ were combined into a single assessment period, t_1_.

Subjects were stratified by the location of their oral tumor. Differences in the baseline results were analyzed using an ANOVA for continuous variables and chi^2^ for categorical variables. A linear mixed-effects model analysis was performed to analyze the longitudinal results of the MTF. Generalized estimation equation analyses were performed to analyze the longitudinal results of TM, SF_tactile_, and SF_thermal_.

A linear mixed-effects model using MAI as the dependent variable was constructed to assess the influence of the measured variables on masticatory performance. Significant factors previously found to influence MAI [4], such as MMO, prosthetic status, number of occlusal units, tumor location, assessment moment, and MBF, were used as fixed effects in the present analysis. TM, MTF, SF_tactile_, and SF_thermal_ were also included as fixed effects. Two-way interactions between the tongue functions and the number of occlusal units and the prosthetic state were included in the model. The last sublevel of each categorical covariate was used as a reference category. Non-significant covariates were removed in a backwards fashion, starting with the interactions. When a significant interaction was present in the model, the main effect was retained in the model (hierarchical models) while the other non-significant main effects were removed in a backward stepwise manner. The statistical analyses were performed using SAS version 9.4 (SAS Institute, Cary, NC, USA).

##### Data availability

The author has full control over the primary data and allows the journal to review the data upon request.

## Results

A total of 123 subjects with a primary oral cavity tumor were included in this study, 30 of whom had a maxillary tumor, 48 had a mandible tumor, and 45 had a tumor in the TFM. The baseline characteristics of these subjects and their functional outcomes are presented in Table 1. The three location groups differed in their mean age, T-stage of the primary tumor, and baseline MAI and MMO values (*p* = 0.036, *p =* 0.014, *p* = 0.021, and *p* < 0.001, respectively). At t_4_, 13 patients treated for a maxillary, 24 treated for a mandible, and 29 patients treated for a TFM tumor were still in the study.

**Table 1:** Baseline characteristics of the included subjects

Categorical variables	Number of individuals (%)			
	Maxilla (*N* = 30)	Mandible (*n* = 48)	TFM (*n* = 45)	*p* value^a^
Gender				
Female	16 (53)	23 (48)	15 (33)	0.179
Male	14 (47)	25 (52)	30 (67)	
Dental status				0.212
Edentulous	7 (23)	13 (27)	5 (11)	
Full dentures	7 (23)	8 (17)	13 (29)	
Full denture vs full denture on implants	0 (0)	2 (4)	4 (9)	
Full denture vs dentate	4 (14)	8 (17)	3 (7)	
Full denture on implants vs dentate	1 (3)	0 (0)	0 (0)	
Full dentures on implants	0 (0)	0 (0)	0 (0)	
Dentate	11 (37)	17 (35)	20 (44)	
Treatment				
Only surgery	12 (40)	24 (50)	23 (51)	0.600
Surgery + radiotherapy	18 (60)	24 (50)	22 (49)	
T-stage				0.000*
T1	5 (17)	14 (29)	23 (51)	
T2	11 (37)	13 (27)	14 (31)	
T3	1 (3)	3 (6)	4 (9)	
T4	13 (43)	18 (38)	4 (9)	
Continuous variables	Mean value (SD)			
	Maxilla	Mandible	TFM	*p* value^b^
Age	68.6 (12.3)	66.7 (12.7)	61.4 (13.0)	0.036*
Number of occlusal units	2.4 (4.1)	2.3 (3.9)	3.8 (5.1)	0.230
Mixing ability index	24.1 (5.9)	23.4 (5.0)	21.0 (4.6)	0.021*
Bite force	223.8 (232.5)	256.5 (329.8)	376.7 (343.7)	0.075
Maximum mouth opening	53.5 (11.8)	46.6 (11.3)	56.0 (9.0)	0.000***

TFM, tongue and floor of the mouth; SD, standard deviation. ^a^, Analyzed using chi^2^ tests for categorical variables; ^b^, analyzed using a one-way ANOVA for continuous variables. *, *p* < 0.05; **, *p* < 0.01; ***, *p* < 0.001

### Tongue force

**Fig. 1:**
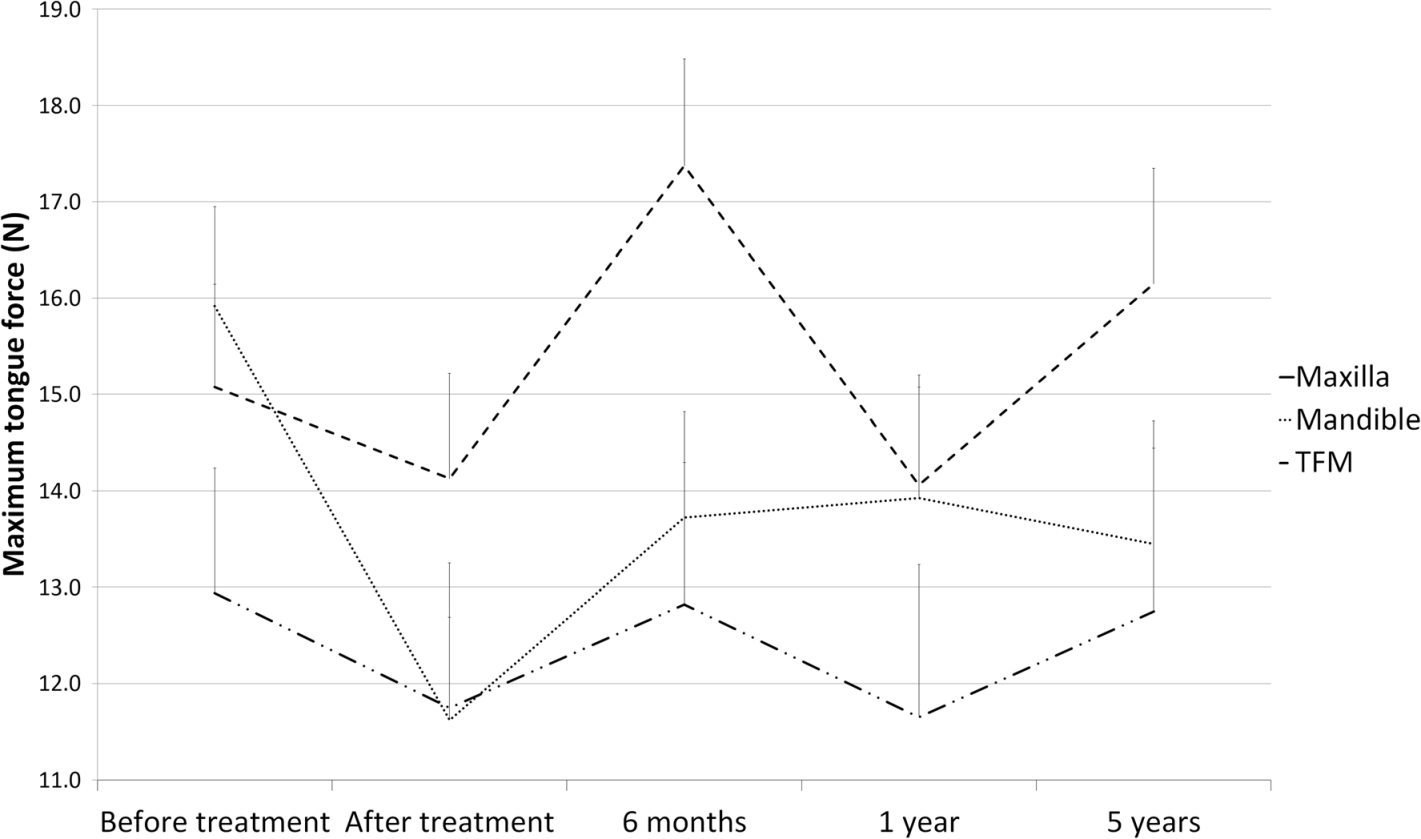
Longitudinal results of maximum tongue force, calculated using a mixed model analysis and presented in Newton. TFM, tongue and floor of the mouth; 6 months; 6 months after treatment; 1 year, 1 year after treatment; 5 years, 5 years after treatment. ‡ and *, *p* < 0.05 between the patients with tumors at the marked locations at the same assessment moment. †, *p* < 0.05 between two assessment moments for patients with tumors in one specific location

At t_0_, the mean MTF was not significantly different between the maxilla, mandible, or TFM groups (Fig. 1). A significant decline in MTF was observed in the mandible group between t_0_ and t_1_ (*p* < 0.001), while in the TFM group, a significant increase in MTF (*p =* 0.003) was observed between t_1_ and t_2_, followed by a significant decrease at t_3_ (*p* = 0.003). The MTF in the TFM group was only significantly higher than those of the maxilla and mandible groups at 6 months posttreatment (t_2_; *p* = 0.014 and *p =* 0.020, respectively).

### Tongue mobility

**Fig. 2:**
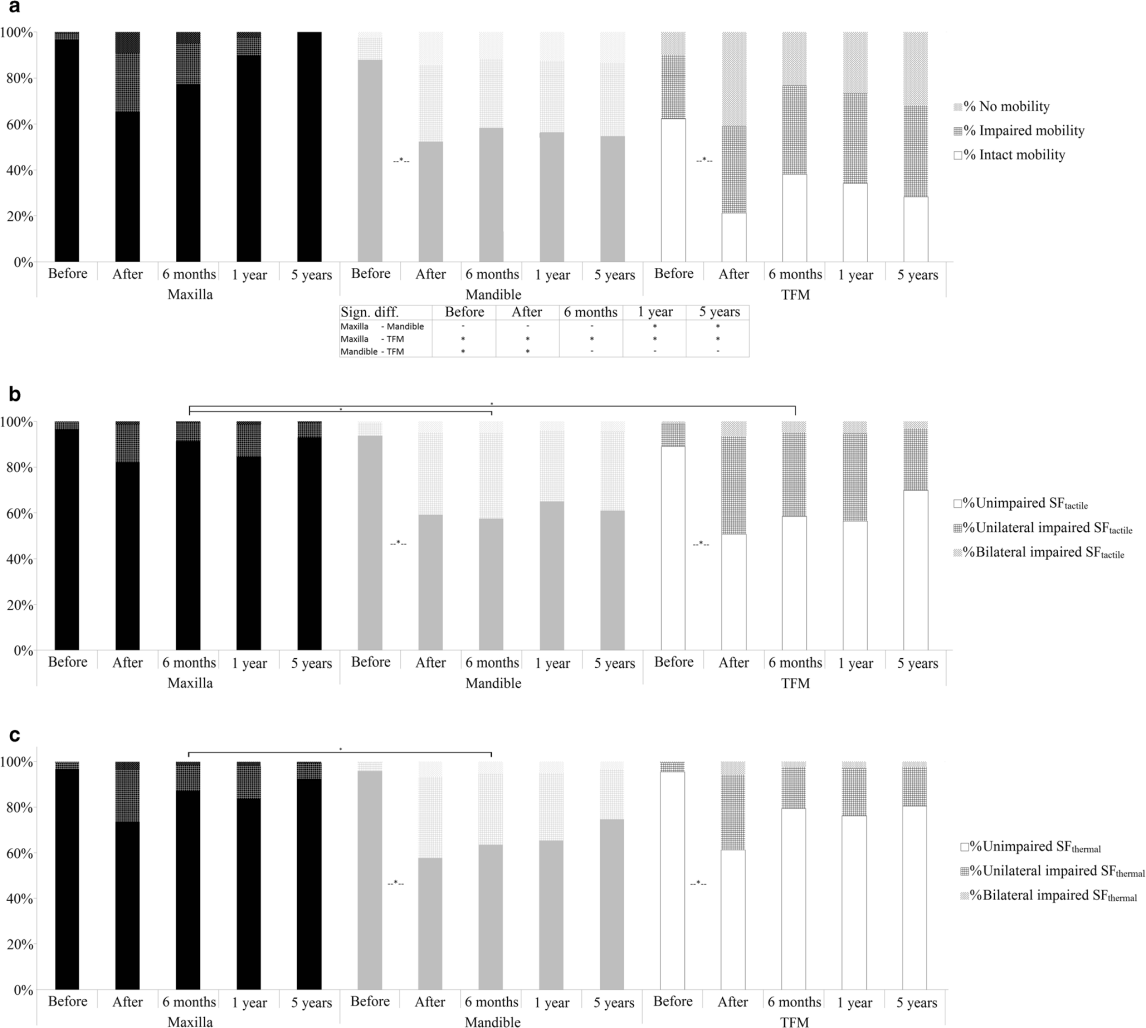
Longitudinal results of tongue mobility (**a**), tactile sensory function (**b**), and thermal sensory function (**c**) in patients with maxillary, mandible, and tongue and floor-of-the-mouth tumors, calculated using a generalized estimation equation analysis. Values are presented as percentages of the total number of patients. Differences between groups were calculated using a chi^2^ test. *, *p* < 0.05. SF_tactile_, tactile sensory function; SF_thermal_, thermal sensory function; TFM, tongue and floor of the mouth; before, 4 weeks before primary treatment; after, 4 to 6 weeks after primary treatment; 6 months; 6 months after treatment; 1 year, 1 year after treatment; 5 years, 5 years after treatment

During the 5-year follow-up, the maxilla group showed no significant change in TM, and at t_4_, all subjects showed unimpaired tongue mobility (Fig. 2a). The changes in the TM of subjects in the mandible group were slightly different to the other groups, in the sense that a significant postoperative decline (89.7 to 52.2%) in the proportion of subjects with unimpaired TM was observed. By t_4_, this proportion had still not been restored (54.6%). The TFM group showed a significantly different distribution of TM levels at t_0_ compared with the patients with maxillary tumors, with only 62.4% having unimpaired TM. At t_4_, only 28.3% of the TFM group showed unimpaired TM, which was significantly different from the maxillary tumor group.

### Tactile sensory function

During the 5-year follow-up, the maxilla group exhibited no differences in tactile sensory function between the various assessment moments, and at t_4_, 92.9% of these patients had an unimpaired tactile sensory function (Fig. 2b). The mandible and TFM groups had a similar distribution of tactile sensory function levels to the maxilla group at t_0_, with 93.6% and 89.0% of these patients exhibiting an unimpaired sensory function, respectively. At t_1_, both the mandible and TFM groups showed a significant decrease in the number of patients with an unimpaired sensory function, to 59.2% and 50.9% respectively. No significant recovery was observed in these groups during the follow-up period, with 61.0% and 69.9% of the mandible and TFM groups, respectively, exhibiting an unimpaired tactile sensory function at t_4_.

### Thermal sensory function

Similar to the tactile sensory function, the thermal sensory function in the maxilla group did not vary between the assessment moments, and at t_4_, 92.4% of the subjects in the maxilla group exhibited an unimpaired thermal sensory function (Fig. 2c). In both the mandible and TFM groups, a significant postoperative decline (t_1_) in the proportion of patients with an unimpaired thermal sensory function was observed, from 95.9 to 57.8% and 95.6 to 64.4%, respectively.

### Masticatory performance and tongue function

The mixed model procedure removed the effects of TM and MMO, because they showed no significant contribution to masticatory performance. The calculated coefficients and standard errors of the mixed model procedure are presented in Table 2. The final model contained the following significant variables: assessment moment, location of tumor, prosthetic state, number of occlusal units, SF_tactile_ score, SF_thermal_ score, MBF, and MTF. Interactions were detected between the assessment moment and tumor location, the prosthetic state and the MBF, the prosthetic state and the MTF, and between the MTF and SF_tactile_, SF_thermal_, and the number of occlusal units.

**Table 2:** The coefficients and interactions derived from the mixed model procedure

	Mixed model	Main effects	SE	Interactions					
	Intercept	26.126	1.799						
				Maxilla	SE	Mandible	SE	TFM	SE
Assessment	Before	2.197	0.735	2.523	1.261	1.494	1.041	0	0
moment	After	4.755	0.759	1.056	1.324	− 0.202	1.067	0	0
	6 months	2.974	0.756	4.170	1.320	2.037	1.073	0	0
	1 year	3.091	0.751	3.333	1.358	2.243	1.097	0	0
	5 years	0	0	0	0	0	0	0	0
Location	Maxilla	− 1.343	1.083						
	Mandible	− 1.031	0.863						
	TFM	0	0						
				MBF	SE	MTF	SE		
Prosthetic state	Edentulous	1.187	1.370	− 0.011	0.009	0.262	0.089		
	Full dentures	1.486	1.584	− 0.023	0.005	0.071	0.110		
	Full dentures vs implant-supported denture	0.001	1.696	− 0.010	0.003	0.102	0.010		
	Full dentures vs dentate jaw	0.888	1.819	− 0.011	0.004	0.028	0.122		
	Implant-supported dentures	12.764	12.645	− 0.058	0.048	0.042	0.515		
	Implant-supported dentures vs dentate jaw	− 6.116	3.181	0	0	0	0		
	Dentate jaws	0		0	0	0	0		
				Occluding pairs			SE		
Thermal sensory function	Unimpaired	− 0.360	1.003	1.6221			0.671		
	Unilateral impairment	− 0.016	1.019	0.7323			0.661		
	Bilateral impairment	0	0	0					
Tactile sensory function	Unimpaired	− 0.650	0.903	− 1.9282			0.867		
	Unilateral impairment	− 0.898	0.940	− 0.9236			0.856		
	Bilateral impairment	0	0	0					
Maximum tongue force		− 0.2817	0.081	0.0230			0.010		
Maximum bite force		− 0.0003	0.001						
Occluding pairs		− 0.518	0.693						

Coefficients and standard errors as calculated using the extended mixed model analysis. The main effects are the coefficients of appropriate for the variable in the row alone. The interactions altering the main effects for each sublevel of the variables are listed in the “interactions” columns. Coefficients of categorical values are multiplied by “1” when true and by “0” when false. Coefficients of continuous variables are multiplied by any fictive outcome of that variable. MBF, maximum bite force; MTF, maximum tongue force; SE, standard error; TFM, tongue and/or floor of the mouth; before, 4 weeks before primary treatment; after, 4 to 6 weeks after primary treatment; 6 months; 6 months after treatment; 1 year, 1 year after treatment; 5 years, 5 years after treatment

**Fig. 3:**
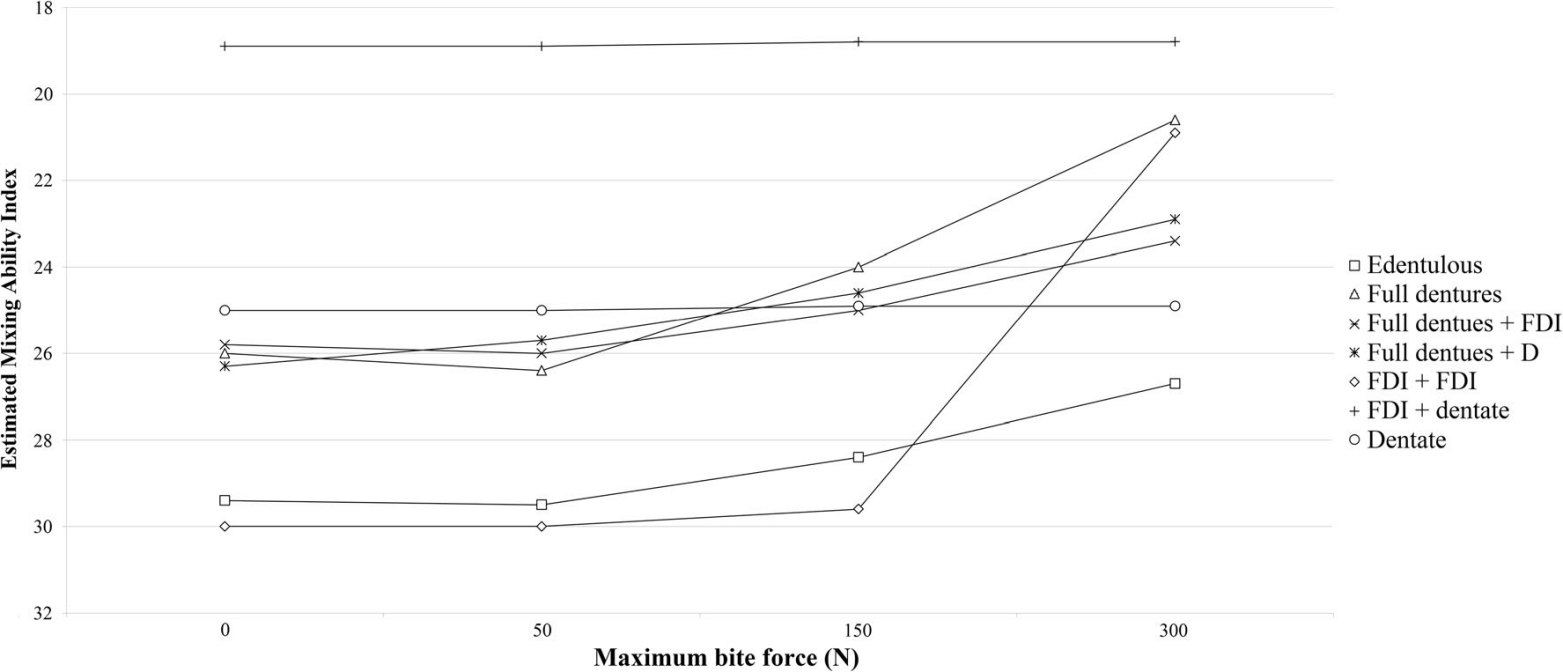
The estimated Mixing Ability Index in patients with different prosthetic states using a bite force of 0 N, 50 N, 150 N, and 300 N, as calculated with the extended mixed model. The remaining conditions are t = 1 year, location = TFM, sensory function = bilateral impairment, tongue force = 15 N, and occlusal units = 0. The bite force was not found to interact with the location of the tumor or the assessment moment, so the influence of the bite force should be similar in the maxilla and mandible groups. *TFM* tongue and floor of the mouth, *FDI* implant-supported full denture, *FD* full denture, *D* dentate jaw

The interactions between the MBF and prosthetic state are visualized in Fig. 3. The effect of MBF on the estimated MAI was greatest in patients with two opposing implant-supported prostheses, followed by those with full dentures, edentulous patients, and those with full dentures opposing a dentate jaw or implant-supported prostheses. No effect was observed for MBF in fully dentate patients nor for those with implant-supported dentures opposing a dentate jaw.

Table 2 is complex due to the large number of coefficients and interactions. Many of these could be visualized similarly to the factors explored in Fig. 3; therefore, we programmed the impact of several factors on the MAI score into an “estimated MAI calculator,” which can be accessed online (Appendix 1). This will allow readers to visualize the effect of variables on the estimated MAI using different characteristics. This is not a predictive tool, nor does it facilitate any clinical applications. It has merely been developed to clearly demonstrate the identified coefficients and interactions of the mixed model.

## Discussion

This 5-year prospective study showed that the tongue force in patients with TFM tumors increased in the 6 months following the treatment, followed by a secondary decline at 1 year after treatment. Patients with TFM or mandible tumors showed a significant postoperative deterioration in function related to TM, SF_thermal_, and SF_tactile_, with the addition of postoperative MTF deterioration in the mandible group. The patients in both the TFM and mandible groups showed no significant improvement in TM, SF_thermal_, or SF_tactile_ during the 5-year follow-up after their treatment. This could be explained by the development of late side effects from the tumor resection and/or the adjuvant radiotherapy, such as tissue fibrosis [17]. A similar longitudinal study of patients with new primary oral and oropharyngeal squamous cell carcinomas also found they had a significant deterioration of tongue sensory function and TM [18]. Transected nerves show little regenerative ability, explaining the absence of sensory recovery. In free-flap reconstruction, this recovery relies mostly on axonal growth in the flap periphery, and nerve anastomosis shows contradictory results [19, 20].

This study did not reveal any impairment of the assessed tongue functions in patients treated for maxillary tumors, because the surgical (and radiotherapy) site required for their treatment did not involve the tongue.

In the mixed model analysis, the effect of the tumor location on masticatory performance was dependent on the moment at which it was assessed. At 5 years after treatment, the TFM group had the worst masticatory performance. In contrast, another cohort study showed that, after 1 year, subjects who had undergone a glossectomy had a better masticatory performance than patients treated with a marginal or segmental mandibulectomy [21].

The effects of tongue force and MBF on masticatory performance varied depending on the prosthetic state of the patients; for example, tongue force had a larger effect on masticatory performance in subjects wearing full prostheses than in subjects where one of the prostheses was implant-supported. The prosthetic state of having two opposing implant-supported full prostheses benefited the most from having a high MBF, showing a poor masticatory performance when their MBF was less than 150 N (Fig. 3). Consequently, if a patient has a bite force lower than 150 N, masticatory performance might not improve as much as desired when committing to full dental rehabilitation. The masticatory performance of subjects with an implant-supported denture opposing a dentate jaw, or two opposing dentate jaws, was barely affected by the MBF.

Several studies underline the importance of bite force for masticatory performance [4, 22–24]. Patients who undergo early dental rehabilitation after implant placement during ablative surgery have been shown to have a significantly higher bite force 5 years after treatment [25], suggesting that early rehabilitation is functionally beneficial. The rates of oral rehabilitation are also generally higher in patients who underwent implant placement during their ablative surgery [26]. In clinical practice, a quick bite force assessment prior to dental rehabilitation would therefore provide an insight into the expected increase in masticatory performance of any rehabilitation, which could be helpful for decision-making [27].

The effects of sensory function and tongue force on masticatory performance were significant, but the magnitude of these effects was influenced by the number of occlusal units present. A higher number of occlusal units was found to decrease both the negative effect of the sensory impairment and the positive influence of a higher MTF on masticatory performance. This is likely caused by the increasing importance of stereognostic ability in patients with fewer occlusal units [22]. In a study of pigs, it was shown that the amplitude of the chewing cycles is altered and that masticatory performance is at risk after sensory dysfunction due to a unilateral lingual nerve transection [28]. Also, a cross-sectional study investigating the relevance of several factors influencing the mixing ability of healthy elderly people highlighted that tongue pressure is more important in denture wearers than for those with natural dentition, which is supported by the coherence in the factors of tongue force and the number of occlusal units [29]. Additionally, edentulous patients (without prostheses) are likely to use their tongue to flatten soft foods against their palates. This necessitates an adequate tongue force for mastication and, vice versa, this use of the tongue might lead to an increase in tongue force [6]. This latter increase is confirmed by a study including healthy subjects, which found a positive correlation between tongue and cheek force and masticatory performance [30]. Maintaining adequate tongue function should thus be attempted by primary reconstruction when possible [8]. Additionally, tongue function could be improved by performing lingual exercises, possibly contributing to the masticatory performance [31].

Surprisingly, the mixed model coefficients suggested that a lower thermal sensory function leads to better masticatory performance; however, the longitudinal results showed equal changes in thermal and tactile sensory function over time, leading to the question of whether these two entities should be analyzed as two separate variables. When the SF_thermal_ and SF_tactile_ coefficients at each level of function are added up, sensory impairment logically caused deterioration in masticatory performance. Thermal and tactile sensory functions are therefore most logically interpreted as a combined sensory function factor in this model.

### Strengths and limitations

The strengths of the present study are the large study population, prospective design, objectively validated assessment methods [11], long follow-up period, and thorough statistical analysis. The relatively large study sample enables the comparison of similar groups of oral cancer patients; however, despite the attempt to classify the patients based on their tumor location, the study groups are somewhat heterogenic. Thus, the extrapolation of these results to a single patient is difficult.

A survivor effect during the 5-year follow-up cannot be ruled out; however, effort has been made to take this into account in the statistical analysis using the mixed model approach [32]. Additionally, the effects of the assessed tongue functions on masticatory performance were not previously analyzed due to the decreasing stability of the obtained mixed model when a large number of factors and interactions are included [4]. Therefore, they were used in the current analysis with less covariates included.

Finally, it should be emphasized that the “Estimated MAI calculator” is not predictive, but merely an illustrative tool for use with the present dataset only. It therefore allows no clinical application.

### Future research

Considering the important aspects of tongue and bite force, future studies should focus on the predictive value of bite force on the functional benefits of dental rehabilitation. Furthermore, the benefits of the preoperative exercising of the tongue and masticatory muscles on masticatory function could be studied. Objective assessments, besides the currently used methods, can include more specific range of motion of the tongue, lateral force measurement, or video fluorography during chewing barium-containing test foods [33]. Using these methods, more specific conditions of adequate tongue functioning related to dental state and mastication can possibly be explored.

## Conclusions

Within the limitations of the present study, the authors conclude that complex interactions exist between the factors influencing masticatory performance in patients treated for oral cancer. An adequate MBF, greater than 150 N, is of great importance for reaching good masticatory performance in the dental implant rehabilitated group. The assessment of bite force during outpatient contact could therefore help in the selection of patients suitable for dental rehabilitation. Finally, the restoration of occlusal units is especially important in patients with sensory deficits and reduced tongue strength.

## Figures and Tables

**Fig. 4:**
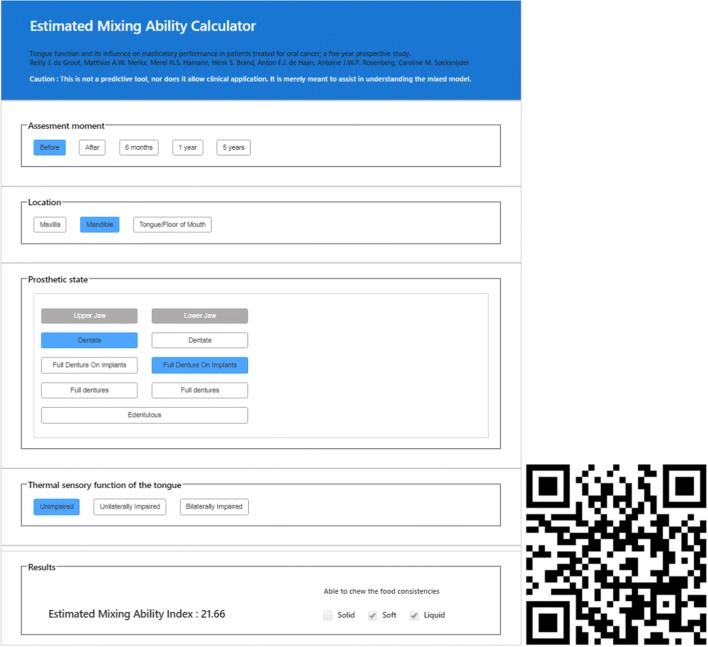
Screenshot of the ‘Estimated MAI calculator’, based on the outcome formula of the mixed model. Hosted on: http://maicalc.azurewebsites.net/ or follow the QR-code
